# Synthesis, adsorption and molecular simulation study of methylamine-modified hyper-cross-linked resins for efficient removal of citric acid from aqueous solution

**DOI:** 10.1038/s41598-020-66592-8

**Published:** 2020-06-15

**Authors:** Xiaoqiang Peng, Pengpeng Yang, Kun Dai, Yong Chen, Xiaochun Chen, Wei Zhuang, Hanjie Ying, Jinglan Wu

**Affiliations:** 1 College of Biotechnology and Pharmaceutical Engineering, Nanjing Tech University, Nanjing, China; 2 National Engineering Technique Research Center for Biotechnology, Nanjing, China; 3 Jiangsu National Synergetic Innovation Center for Advanced Materials, Nanjing, China; 4 State Key Laboratory of Materials-Oriented Chemical Engineering, Nanjing, China

**Keywords:** Biochemistry, Biotechnology

## Abstract

A series of methylamine-modified hyper-cross-linked resins were fabricated from chloromethylated polystrene-co-divinylbenzene by two continuous reactions (Friedel-Crafts alkylation and amination). The BET surface area and pore volume of the as-prepared resins took a positive correlation to the reaction time and temperature during alkylation reaction while lessened during amination process. When running batch adsorption experiments for adsorption of citric acid, the methylamine-modified resin named HM-65-2 showed higher adsorption capacity of 136.3 mg/g and selectivity of 6.98 (citric/glucose) than the precursor resins. The pseudo-second-order rate model fitted better than the pseudo-first-order model, implying the adsorption sites distributed on the resins surface tended to be heterogeneous. Subsequently, the interactions between citric acid and the resin were investigated by means of molecular simulation. Simulation result showed the addition of nitrogen-containing groups significantly enhanced the adsorption performance of citric acid. Lastly, the dynamic column experiments were performed to obtain the suitable operating conditions for the citric acid adsorption.

## Introduction

Citric acid (CA, 2-hydroxypropane-1,2,3-tricarboxylic acid, C_6_H_8_O_7_), as an important organic acid associated with thermoplastics fields in virtue of versatility, plays multiple roles in food, pharmaceutical, chemical or bio-processing industry etc. due to its favorable physico-chemical properties^1^. Citric acid is mainly manufactured by the fermentation of carbohydrates by Aspergillus niger using submerged processes, which is relatively easy to achieve high yield and environmental friendly product^2^. However, the recovery of citric acid from the fermentation broth is not straightforward. The conventional separation technique used in industry includes several batch steps increasing the production cost and generating a considerable amount of environmentally harmful waste^3^. Some novel separation techniques, i.e., solvent extraction^4^, electro-dialysis^5^, membrane separation^6^, ion exchange^7^ and adsorption^8^ have been proposed for citric acid recovery and purification, among which adsorption has been considered to be one of the most efficient and simple method due to its wide application scope, energy-saving, and environmentally friendly. Most adsorbents selected for recovering citric acid are based on weak-base ion-exchangers^9^, which needs some harsh desorption conditions i.e. alkaline solutions and limits its practical application. Gluszcz *et al*.^10^ studied the adsorption behaviors of different ion exchange resins for citric acid recovery from aqueous solutions and ascertained that the resin with a tertiary amine functional group was suitable for citric acid recovery. Juang *et al*.^11^ explored the adsorption behaviors of citric acid on macroporous resins impregnated with tri-n-octylamine. Other adsorbents including hydroxyapatite^12^, oxides^13^ and chitosan microspheres^14^ have been tried for recovery of citric acid from aqueous solution, but the low adsorption capacity and complex desorption process limited their further application. Related literature reports were summarized in Table 1, which some adsorption amounts data were calculated based on the batch adsorption data at 298 K. It can be seen that the adsorption capacity of citric acid by ion exchange resins were generally higher than that of these non-ionic adsorbents, but excessive adsorption amounts usually causes more difficulties in desorption process. Therefore, it is beneficial to find a suitable adsorbent with moderate adsorption capacity of citric acid. Fortunately, the hyper-cross-linked poly(styrene-co-divinylbenzene) (PS-DVB) resins, as a typical adsorbent, possessing abundant microporous or mesoporous structure, have attracted substantial attention for the potential separation of organic components owe to its outstanding thermal stability, moderate surface polarity, and high specific surface area.

**Table 1:** Literatures list of citric acid adsorption by some typical adsorbents.

Name of adsorbents	Type of adsorbents	Adsorption capacity of citric acid (mg/g)
330 resin	Weakly basic ion exchange resin	76.4810^7^
Amberlite IRA-67	Weakly basic ion exchange resin	316.1^10^
XAD-16	Macroporous resin	126.72^11^
/	Hydroxyapatite	19.296^12^
RCM	Chitosan microsphere	77.0^14^

The hyper-cross-linked polystyrene resins proposed by Davankov *et al*.^15^ are usually synthesized from linear polystyrene or low cross-linked polystyrene by adding bi-functional/multifunctional cross-linking organic reagents such as tetrachloromethane(CCl4), dichloroxylene(DCX)^16^, trifunctional tris-(chloromethyl)-mesitylene(TCMM) and the Friedel–Crafts catalysts including anhydrous zinc chloride, iron (III) chloride and stannic (IV) chloride^17^. In order to avoid using certain ether carcinogens, the hyper-cross-linked resins can also be prepared from macroporous low cross-linked chloromethylated polystyrene by Friedel–Crafts alkylation reactions. Hence, the obtained hyper-cross-linked networks consist of an intensive bridging of strongly solvated polymer chains with conformationally rigid links, leading a major shift of their pore diameter distribution from predominate mesopores to mesopores–micropores bimodal distribution. This also resulted a notable amplify of the Brunauer–Emmet–Teller (BET) surface area and pore volume. Because of these significant changes, the hyper-cross-linked polystyrene resins display excellent adsorption capacities towards non-polar and weakly polar compounds from aqueous solutions. However, the weakness of low capacity to polar substances restrains the applications of hyper-cross-linked resins. In order to enhance the adsorption capacity of polar compounds, the resins can be introduced into some polar comonomers^18^. Moreover, on the purpose of the chemical modification introduced into the resin surfaces, the functionalized hyper-cross-linked resins can also be prepared from the chloromethylated polystyrene according to two continuous procedures. The first produce is that the chloromethyl groups react with another neighboring phenyl ring of the polystyrene by formation of an equivalent number of diphenylmethane-type rigid polymer bridges during a typical Friedel–Crafts reaction, and the second produce is the reaction between the residual chloromethyl groups of the hyper-cross-linked resins and then the functional groups introduced. The residual chloromethyl content of the polymer obtained after the first produce reaction determines the resin pore structure and the amount of subsequent functional-group modification. Thus, it is feasible to adjust the hyper-cross-linked resin morphology and polarity by ordinarily governing the corresponding Friedel–Crafts reaction parameters. Although numerous references have shown that the resins modified with polar functional groups (i.e., amino group) exhibit predominant adsorption capacity to certain weak acids, such as p-aminobenzoic acid^19^, salicylic acid^20^, phenol^21^, and 2-naphthol^22^ due to the high BET surface area providing more active sites, there are relatively few reports concerning the adsorption of citric acid on the methylamine-modified hyper-cross-linked resins.

In the present work, six kinds of the hyper-cross-linked resins were synthesized from the commercial available macroporous cross-linked chloromethylated polystyrene resins by regulating the Friedel–Crafts reaction time (1.0, 2.0 and 4.0 h) and reaction temperature (65 and 85 °C). Then these hyper-cross-linked resins were chemically modified by superfluous methylamine reagent to obtain the methylamine-modified resins. Thereafter the adsorption capacity and selectivity of the resins toward citric acid/glucose were evaluated by the batch adsorption experiments, among which one named HM-65-2 was selected as the proper resin to adsorb citric acid from aqueous solution. Afterwards, the interaction energy between citric acid and resins (unmodified or methylamine-modified resins) were analyse by means of density functional theory (DFT) based on B3LYP mode. The result of theoretical computation proved methylamine-modified resins superiority in adsorbing citric acid due to stronger interaction effect, which provides the fundamental information for the future applications.

## Experimental Section

### Materials

Macroporous low cross-linked chloromethylated polystyrene used as a reaction precursor was purchased from Nankai University Chemical Plant (Tianjin, China). Anhydrous iron (III) chloride, 1, 2-dichloroethane (DCE), methylamine and ethanol were all analytical reagents and used without further purification. Glucose and citric acid (purity ≥ 99%) purchased from Merck (Darmstadt, Germany) were employed as adsorbates in this work.

### Synthesis of methylamine-modified hyper-cross-linked polystyrene resins

**Figure 1:**
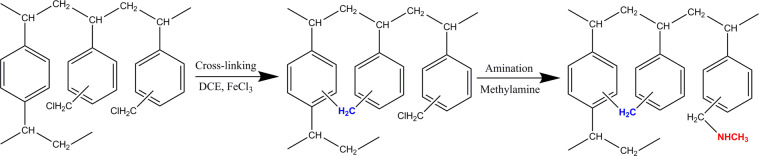
Synthetic procedure of the methylamine-modified hyper-cross-linked resins.

As shown in Fig. 1, the methylamine-modified hyper-cross-linked polystyrene resins were prepared by two continuous steps and these steps were derived from ref. ^23^ except for different reagents. In a typical synthetic procedure, under mild mechanical stirring, 150 mL of the 1, 2-dichloroethane (DCE) was applied as the solvent to swell sufficiently 15 g of the macroporous cross-linked chloromethylated polystyrene at room temperature for 24 h and then 3 g of the anhydrous iron (III) chloride was added used as a catalyst. After refluxing mixture for various time (1 h, 2 h and 4 h) at set temperature (65 and 85 °C) controlled by thermostatic oil bath, the obtained hyper-cross-linked polystyrene resins were named as H-65-1, H-65-2, H-65-4, H-85-1, H-85-2 and H-85-4. After rinsing completely, the polymeric beads above were mingled with superfluous methylamine reagent kept at 323 K for 20 h, and then the formed spherical particles were washed with deionized water until neutral pH, followed extracted with ethanol in Soxhlet apparatus for 10 h and then dried under vacuum at 323 K for 12 h. The methylamine-modified hyper-cross-linked polystyrene resins prepared were labeled as HM-65-1, HM-65-2, HM-65-4, HM-85-1, HM-85-2 and HM-85-4.

### Characterization of the resins

The Brunauer-Emmett-Teller (BET) surface area and pore volume of the resins prepared were determined by the N_2_ adsorption-desorption isotherms using a Micromeritics ASAP 2020 Physisorption Analyzer (Micromeritics, the United States) with the temperature at 77 K and the results were calculated with the relative pressure (P/P_0_) at 0.05~0.35 according to the adsorption isotherms. The elemental analysis of resin was performed by a VarioEL Elemental Analysis System and the chlorine content of the resins was received as:

1 $$Cl( \% )=100-C( \% )-H( \% )-N( \% )-O( \% )$$

The Fourier-transformed infrared (FT-IR) spectra of the resins were collected on a Nicolet 510 P FT-IR instrument (Nicolet, the United States) via the potassium bromide technique with the wavenumbers ranged from 500 to 4000 cm^−1^.

### Theoretical computation

Theoretical computation were carried out with the Materials Studio software 7.0 (BIOVIA, CA) on a P4-2.6 G computer in this part. The assumed complexes between the citric acid and the simplified structure of resins (including unmodified and methylamine-modified resins) were optimized by means of density functional theory (DFT) based on B3LYP mode. The interaction energy (ΔE, kJ/mol) of different system was introduced according to Eq. (2)

2 $$\Delta E={E}_{Total}-({E}_{CA}+{E}_{re\sin })$$

where E_CA_ (kJ/mol) is the total energy of citric acid molecular structure, E_resin_ (kJ/mol) is the total energy of resins structure and E_Total_ is the total energy of CA-resin structure. Generally, the larger ΔE value means higher energy barrier as well as weaker affinity between the dsorbate and adsorbent^24^.

### Batch adsorption experiments

The loading and selectivity of the resins prepared for citric acid and glucose adsorption were evaluated to screen a suitable adsorbent used in the following experiments. In a typical adsorption experiment, 2 g of the tailor-made resins were weighed accurately in a series of flasks with a stopper and then mixed with 10 mL of solution containing 100 g/L citric acid and 10 g/L glucose. Then the flasks were shaken in a water-bath thermostatic oscillator (the agitation speed was set to be 200 rpm) for 4 h at 298 K to guarantee the equilibrium of adsorption process. Subsequently, the resins were filtered and the equilibrium concentration (C_e_ (g/L)) of citric acid and glucose in the solution were determined and the equilibrium adsorption capacity (q_e_ (mg/g)) and selectivity coefficient (α) for the citric acid and glucose were obtained as the following equations^25^:

3 $${q}_{i,e}=\frac{({C}_{i,0}-{C}_{i,e})V}{W}$$

4 $$\alpha =\frac{{q}_{i,e}/{C}_{i,e}}{{q}_{j,e}/{C}_{j,e}}$$

where i, j represent the citric acid and glucose adsorbates, q_e_ is equilibrium adsorption capacity (mg/g), C_0_ and C_e_ are the initial and the equilibrium concentration of adsobate (g/L), V is the volume of the solution (mL) and W is the weight of the resins (g).

Comparison of the results of q_e_ and α, one of the resins was selected as the proper resin to separate citric acid and glucose. The citric acid adsorption isotherms on the selected resin were performed at three different temperatures (298, 308 and 318 K). 2 g of the resins was completely dispersed in 10 mL of citric acid aqueous solution with the concentration range from 15 to 150 g/L. Then the solutions were continuously shaken in a thermostatic oscillator with identical rotational speed (200 rpm) for 4 h at a desired temperature to ensure the adsorption equilibrium.

The kinetic adsorption of citric acid on the selected resin was performed by analyzing the uptakes on the resin until the equilibrium was reached. 80 g of the resins and 400 ml of citric acid solution with the initial concentration (100 g/L) were quickly introduced into a 500 mL round-bottomed flask. The mixture was continuously stirred at 298 K and 0.5 mL of solution was sampled at different time intervals. The concentration of the residual solution was determined and the adsorption capacity at the contact time t was calculated as^19^:

5 $${q}_{t}=\frac{({C}_{0}-{C}_{t})V}{W}$$

where q_t_ (mg/g) and C_t_ (g/L) are the adsorption capacity and the concentration at contact time t (min).

### Column breakthrough experiments

The column breakthrough experiments were performed to better reflect the adsorption behavior of citric acid on the resin. In a typical dynamic experiment, 120 g of wet resins were packed in a glass column. The concentration of citric acid from the effluent was dynamically recorded until it reached the initial concentration. The breakthrough point (C_t_/C_0_ = 0.05) and saturated point (C_t_/C_0_ = 0.95) was measured and the breakthrough capacities (q_b_, mg/g) and saturated capacities (q_s_, mg/g) of citric acid on the resins were estimated by the following equations^26^:

6 $${q}_{b}=\frac{Q{C}_{0}}{W}{\int }_{0}^{{t}_{b}}\,\left(1-\frac{{C}_{t}}{{C}_{0}}\right)dt$$

7 $${q}_{s}=\frac{Q{C}_{0}}{W}{\int }_{0}^{{t}_{s}}\,\left(1-\frac{{C}_{t}}{{C}_{0}}\right)dt$$

where Q is the elution flow rate (mL/min), t_b_ and t_s_ are the breakthrough time and saturated time (min).

### Analytical method

The concentrations of citric acid and glucose in an aqueous solution were determined by HPLC equipped with a HPX-87H ion exclusion column (300 mm × 7.8 mm, Agilent Technologies, America) and a differential detector (G1362A, Agilent Technologies, America) at a flow rate of 0.6 mL/min. The mobile phase chosen 5 mmol/L H_2_SO_4_ aqueous solution and temperature was set at 55 °C. When the concentration was higher than 10 g/L, the sample was diluted before detected using HPLC. (The standard curves and chromatogram of citric acid and glucose were shown in Fig. S1 and S2 in the **Supplementary materials file**)

## Results and discussion

### Characterization of methylamine-modified hyper-cross-linked resins

**Figure 2:**
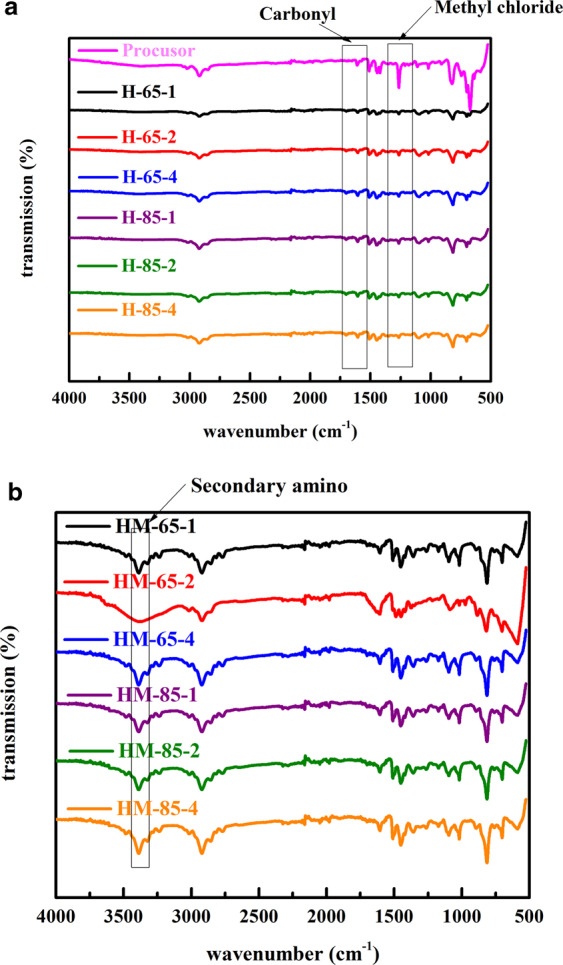
Infrared spectra of (**a**) hyper-cross-linked resins unmodified under different parameters, (**b**) hyper-cross-linked resins modified by methyamine under different parameters.

The FT-IR spectra of the hyper-cross-linked unmodified resins (H-65-1, H-65-2, H-65-4, H-85-1, H-85-2 and H-85-4) and methylamine-modified resins (HM-65-1, HM-65-2, HM-65-4, HM-85-1, HM-85-2 and HM-85-4) are indicated in Fig. 2. Comparing with precursor resins, there was a new moderate adsorption band located at 1705 cm^−1^ assigned to the carbonyl groups stretching resulting from oxidation of chloromethyl groups after the Friedel-Crafts reaction in the hyper-cross-linked resins^27^. Simultaneously, the strong vibration of the -CH_2_Cl groups (at 1265 cm^−1^) was greatly weakened^28^, which in accordant with the sharp decrease of the chlorine content shown in Table S2. These results implied that the -CH_2_Cl groups were consumed and transformed to rigid cross-linking bridges after the Friedel-Crafts reaction. After the amino reaction, there appeared a strong and wide vibrational band at 3417 cm^−1^ representing the N-H stretching from the secondary amino in the amino-modified resins^29^, which confirms the successful uploading of methylamine on the hyper-cross-linked resins.

Fig. S3(a,c) display the N_2_ adsorption-desorption isotherms of unmodified resins reacted at 65 °C, while Fig. S3(b,d) were the ones reacted at 85 °C. Comparing with the precursor resins, the N_2_ uptake of the obtained hyper-cross-linked resins are extremely larger at the same relative pressure, indicating Friedel-Crafts reaction contributes to the porous addition. At a relative pressure below 0.05, the N_2_ uptakes increased sharply with increasing of the relative pressure, and at a relative pressure above 0.95, there was an increase of the N_2_ uptakes as well. According to the IUPAC classification^30^, these isotherms nearly closed to type-II, which demonstrated that the micropores were predominant and the macopores were also existent. Meanwhile, visible hysteresis loops of the desorption isotherms indicated the existence of a few mesopores. However, after the amination reaction, the decrease of mesopores led to the reduce of the BET surface area of the resins (Table S2), which was in agreement with the conclusion reported in ref. ^31^.

The BET surface area of the precursor resin was only 31.88 m^2^/g, while abundant porous structure were produced after the Friedel-Crafts reaction, causing a significant increase of the BET surface area and pore volume. This increase may be assigned to contribute from the formation of the methylene cross-linking bridges in the Friedel-Crafts reaction. Longer reaction time, as well as higher reaction temperature, would bring about more cross-linking bridges^32^. While methylamine-modified resins exhibited significantly decrease of the BET surface area and pore volume, which may be from the partial hole collapse during the polar modification process^22,31^. Furthermore, this phenomenon could also be explained with an increased modified solvent when uploaded with methylamine^33^. During the amination process, the original mesoporous structures in the resin were filled with methylamine molecules and formed some closed pore structures. The combined effect of pore collapse and modifiers made the surface areas of the final methylamine-modified resins were smaller than that of the hyper-cross-linked resins.

### Comparison of resin adsorption properties

**Figure 3:**
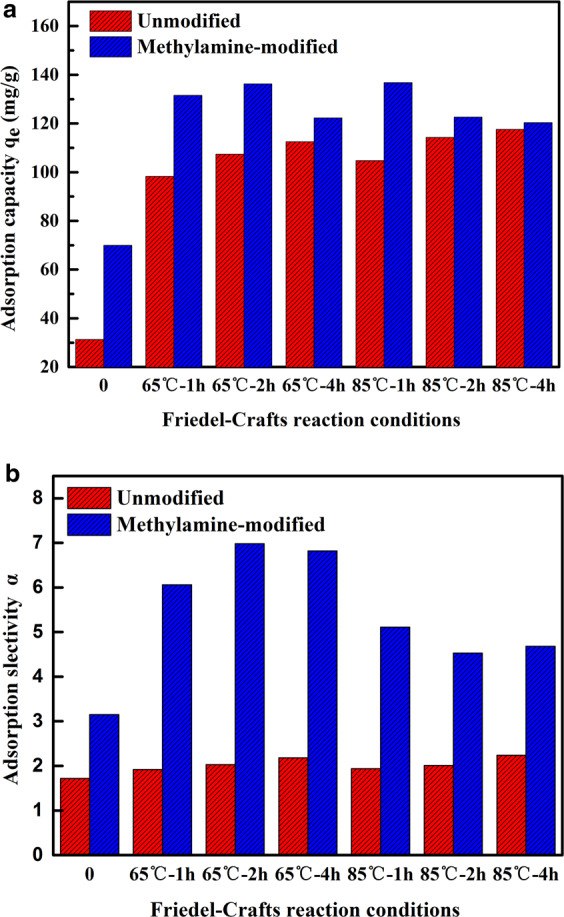
(**a**) Adsorption capacity of citric acid and (**b**) the selectivity of citric acid.

Figure 3(a,b) reveal the adsorption capacity (q_e_, mg/g) and selectivity of citric acid/glucose (α) onto the unmodified resins and methylamine-modified resins. The adsorption capacity of citric acid is proportional to the Friedel-Crafts reaction time and temperature for the unmodified resins, indicating more favorable binding sites were formed as the increase of reaction time and temperature. Whereas the adsorption selectivity of citric acid and glucose onto the unmodified resins appears were insensitive to the reaction time and temperature. After the methylamine modification, the q_e_ and α value of the modified resins exhibit sharp increase comparing to the unmodified resins, and the reaction temperature and time also have effect for this increase. Although the BET surface area possessing numerous adsorption sits significantly reduced after methylamine-modified, the introduced amino groups would provide other stronger interactions, such as hydrophobic interaction, electrostatic interaction or hydrogen bonding^34^. These stronger interactions are sufficient to enhance the adsorption capacity and selectivity of citric acid. Comparing the results depicted in Fig. 3, the methylamine-modified resins named HM-65-2, occurring Friedel-Crafts reaction at 65 °C during 2 h, showed higher adsorption capacity of 136.3 mg/g and selectivity of 6.98 (citric/glucose) than others. Consequently the HM-65-2 resin was selected to complete the other adsorption experiments for removing citric acid from aqueous solutions.

Furthermore, a compare experiment of commercial weak basic anion-exchange resin (Amberlite FPA53) was carried out to illuminate the superiority of the tailor-made methylamine-modified resin (more experimental details can see in Supplementary materials). Results shown in Fig. S4 indicated that the adsorption capacity of citric acid on the weak anion-exchange resin reached the maximum value (378.1 mg/g), which was higher than that of HM-65-2 resin (136.3 mg/g). But the desorption rates (<40%) were lower than that of HM-65-2 resin (>80%) when only using hot water (65 °C) as an eluent. This enormous difference can be attributed to the stronger acid-base interaction between the ion-exchange resin and citric acid. Higher desorption rate presented the superiority of the tailor-made HM-65-2 resin, that is, a benign separation routine may be developed to purify citric acid from its fermentation broth by using only hot water as an eluent in future.

### Results of molecular simulation

**Figure 4:**
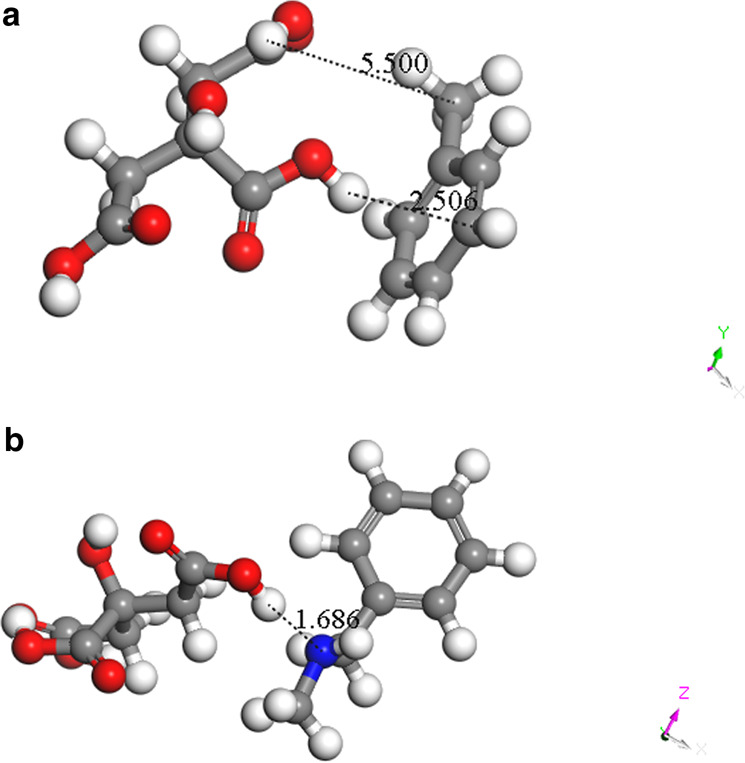
Complexes of (**a**) the CA-unmodified resin and (**b**) CA-methylamine-modified resin.

Figure 4 presents complexes of the CA-unmodified resin and CA-methylamine-modified resin. It could be seen that the carboxyl of citric acid was biased toward the nitrogen-containing group of the resin after methylamine modification while there was not of the unmodified resin. Calculated interaction energy shown in Table 2 also proved this. The interaction energy of CA-methylamine-modified resin was smaller than that of CA-unmodified resin, including that the citric acid was preferentially adsorbed onto the methylamine-modified resin and this preference could attribute to the amino group with stronger electrostatic attraction.

**Table 2:** Calculated interaction energy of (a) the CA-unmodified resin and (b) CA-methylamine-modified resin.

	E_CA_ (×10^6^ kJ/mol)	E_resin_ (×10^6^ kJ/mol)	E_Total_ (×10^6^ kJ/mol)	ΔE (kJ/mol)
(a)	−2.107	−0.757	−2.864	−170.7
(b)	−2.107	−1.019	−3.130	−3396.0

### Adsorption isotherms

**Figure 5:**
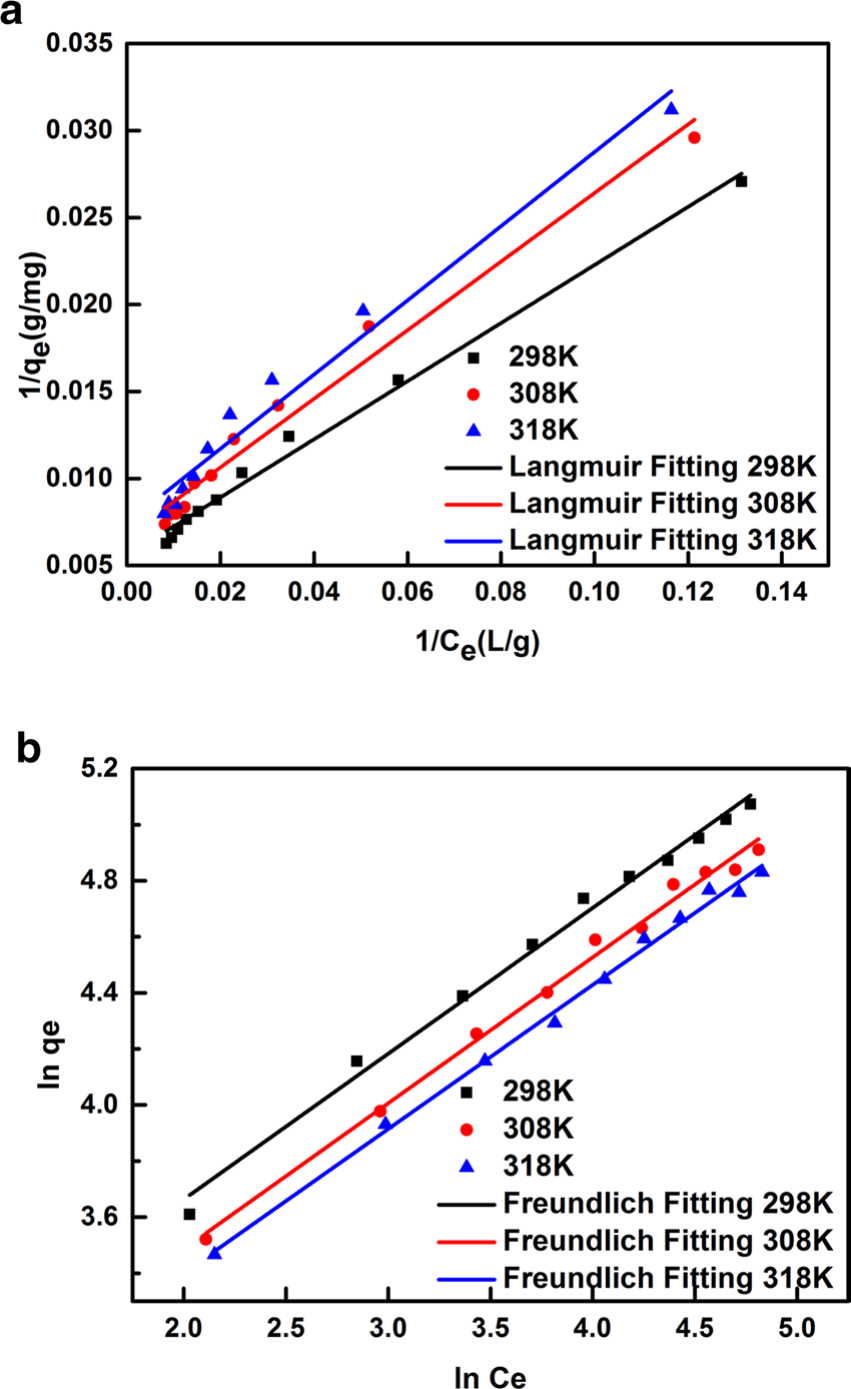
The adsorption isotherm (a: Langmuir model and b: Freundlich model) of citric acid adsorbed on HM-65-2-1 resin from aqueous solution at 298 K, 308 K, 318 K, respectively.

Figure 5 shows the adsorption isotherms of citric acid on the HM-65-2 resins from aqueous solution with temperature of 298, 308 and 318 K. It could be clearly observed that the adsorption capacity took a positive correlation to the equilibrium concentration and a negative correlation to the temperature, implying that the adsorption was an exothermic process. Furthermore, two typical isotherm models, the Langmuir and Freundlich models were used to analyze the experimental data. Two equations are described by linear as:

8 $${\rm{Langmuir}}\,{\rm{model}}:\frac{1}{{q}_{e}}=\frac{1}{a{C}_{e}}+\frac{1}{b}$$

9 $${\rm{Freundlich}}\,{\rm{model}}:\,\mathrm{ln}\,{q}_{e}=\,\mathrm{ln}\,{K}_{F}+\frac{1}{n}\,\mathrm{ln}\,{C}_{e}$$

where C_e_ is the equilibrium concentration in the solution (g/L), q_e_ is the equilibrium adsorption capacity of citric acid onto the resins (mg/g), and a [(mg L)/g^2^], b (mg/g), K_F_ [(mg/g)(L/mg)^1/n^] as well as n are the characteristic constants.

The fitting curves are shown in Fig. 5 and the corresponding parameters a, b, K_F_ and n, as well as the correlation coefficients R^2^ by the linear method are summarized in Table 3. It reveals that both Langmuir and Freundlich equations are suitable for prediction of the adsorption data since R^2^ > 0.970. The Freundlich isotherm appears to be more appropriate than the Langmuir isotherm due to the higher R^2^. The larger characteristic constants (n > 1) indicating the preferential nature of this adsorption process infer that the adsorption sites distributed on the resins surface tends to be heterogeneous because of the polar modification^35^. Furthermore, in the Langmuir model, b is related to the bonding energy coefficient^36^. As the temperature increases, the b value becomes smaller, indicating the force between the adsorbent and the adsorbate decreases, which testified its exothermic.

**Table 3:** Langmuir and Freundlich isotherm-model parameters of citric acid onto the HM-65-2 resin.

T(K)	Freundlich			Langmuir		
	K_F_	n	R^2^	a	b	R^2^
298	13.79	1.923	0.9914	5.987	179.5	0.9912
308	11.54	1.922	0.9926	5.072	149.5	0.9758
318	10.73	1.946	0.9948	4.690	134.4	0.9709

Some important thermodynamic basic parameters such as the Gibbs free energy change (ΔG, kJ/mol), adsorption enthalpy (ΔH, kJ/mol), and adsorption entropy (ΔS, J.mol^−1^.K^−1^) are obtained according to the following expressions

10 $$\Delta G=-\,RT\,ln\,{K}_{C}$$

11 $$\Delta G=\Delta H-T\Delta S$$

where R is the universal gas constant (8.314 J.mol^−1^.K^−1^), T is the solution temperature (K), and K_C_, the thermodynamic equilibrium constant, can be determined by plotting ln (C_s_/C_e_) versus C_s_ and extrapolating C_s_ to zero^37^.

The ΔG for the adsorption of citric acid on the HM-65-2 resins can be attained from substitution calculation and the ΔH and ΔS values figured out according to the slope and intercept of plotting ΔG versus T are tabulated in Table 4. It is evident that ΔH (−7.792 kJ/mol) is negative ignoring the influence of temperature, suggesting that citric acid adsorption on HM-65-2 resin is exothermic process and the adsorption is not solely simple physical process while it may involves a weakly bonding force formation^38^. The negative ΔG at all temperature are resulted from the spontaneous nature of the adsorption process on resin polar modified and the positive ΔS (20.35 J.mol^−1^.K^−1^) indicates the increase slightly of system randomness at the solid-solution interface during adsorption^39^.

**Table 4:** Thermodynamic parameters of citric acid adsorbed onto the HM-65-2 resin.

T (K)	K_C_	ΔG (kJ/mol)	ΔH (kJ/mol)	ΔS (J.mol^−1^.K^−1^)
298	2.027	−1.751	−7.792	20.35
308	1.813	−1.524		
318	1.663	−1.345		

### Adsorption kinetics

**Figure 6:**
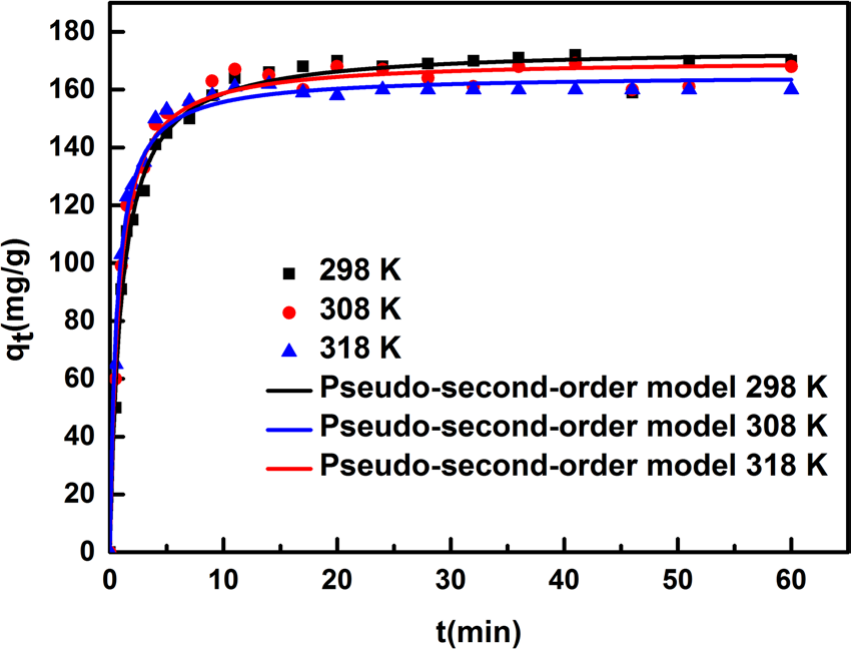
The adsorption kinetic curve of citric acid adsorbed on HM-65-2-1 resin from aqueous solution at 298 K, 308 K, 318 K, respectively.

Figure 6 displays the kinetic curves of citric acid adsorption on the HM-65-2 resin from aqueous solution at 298, 308 and 318 K. The samples had a relatively fast adsorption rate in the initial minutes followed by a raised plateau, implying that the methylamine-modified resins display an excellent kinetic property for citric acid and suggesting that the process likely happens through intermolecular forces^40^. Meanwhile, the required time for the equilibrium was gradually shorter with increasing temperature, suggesting that a higher temperature induces a faster adsorption rate. This phenomenon might owe to the increasing mobility of citric acid and the number of molecules with sufficient energy under higher temperature, and they were more easily undergo an interaction with the active sites at the resin surfaces.

**Figure 7:**
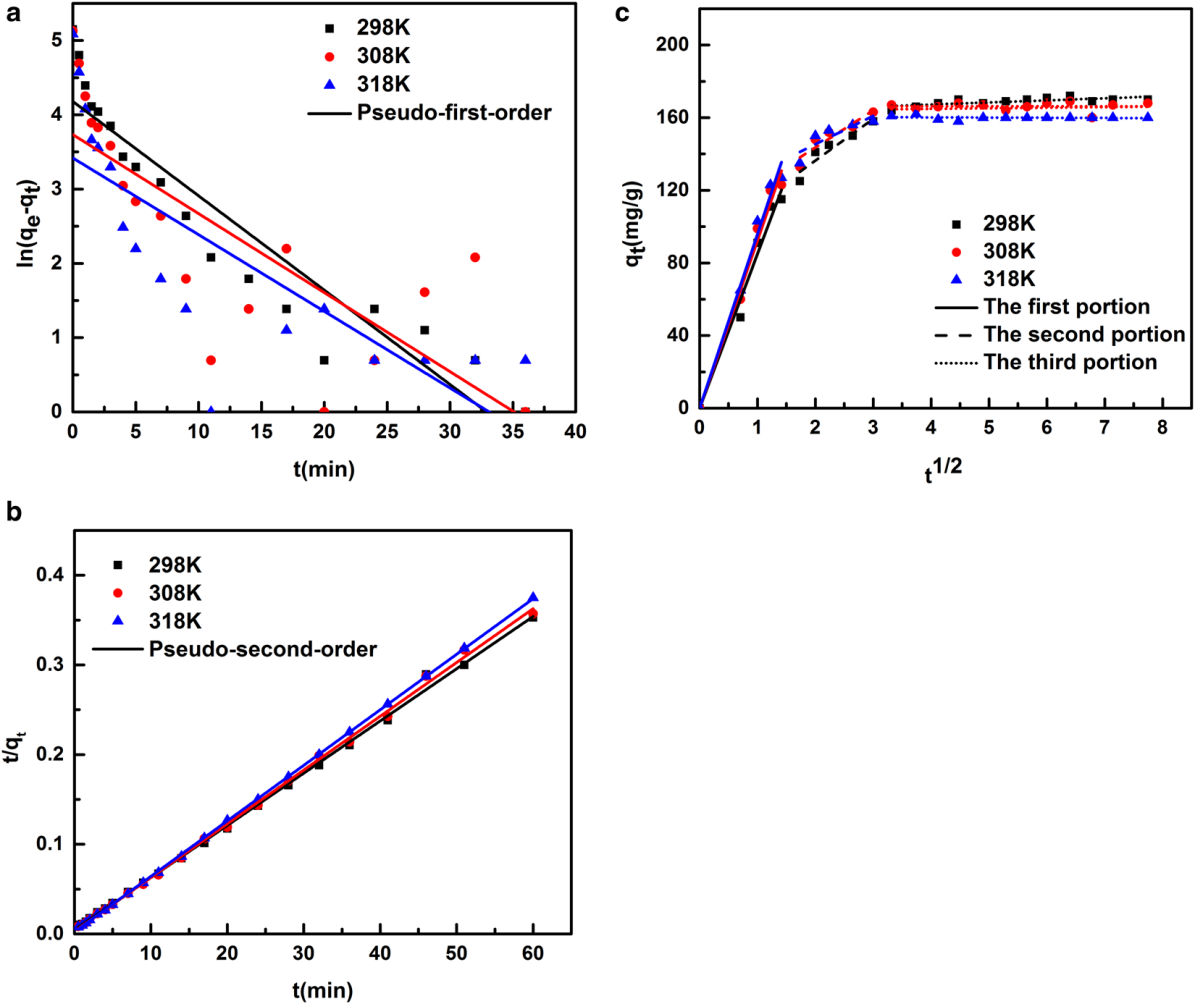
Linear correlations of different models fitting adsorption kinetic data: (**a**) pseudo-first-order model; (**b**) pseudo-second-order model; (**c**) Weber-Morris model.

In order to study the mechanism of the adsorption kinetic process, three commonly adsorptions kinetic models, the pseudo-first-order model, pseudo-second-order model and Weber-Morris model were used to fit the kinetic data (seen Fig. 7), equations are shown as follows:

12 $${\rm{Pseudo}} \mbox{-} {\rm{first}} \mbox{-} {\rm{order}}\,{\rm{model}}:\mathrm{ln}({q}_{e}-{q}_{t})=ln\,{q}_{e}-{K}_{1}t$$

13 $${\rm{Pseudo}} \mbox{-} {\rm{second}} \mbox{-} {\rm{order}}\,{\rm{model}}:\frac{t}{{q}_{t}}=\frac{1}{{K}_{2}{q}_{e}^{2}}+\frac{t}{{q}_{e}}$$

14 $${\rm{Weber}} \mbox{-} {\rm{Morris}}\,{\rm{model}}:{q}_{t}={K}_{3}\sqrt{t}+C$$

where K_1_ (min^−1^), K_2_ (g.mg^−1^.min^−1^) and K_3_ (mg.g^−1^.min^−1/2^) are the constants of pseudo-first-order, pseudo-second-order and Weber-Morris models, respectively. According to the corresponding model parameters and the correlation coefficient (R^2^) shown in Tables 5 and 6, the adsorption kinetic data could be fitted well by the pseudo-second-order model, and the calculated equilibrium adsorption capacities are relatively close to the real experimental results, suggesting the chemisorption would be the rate-control mechanism^41^.

**Table 5:** Coefficients of the pseudo-first-order model and pseudo-second-order models at different temperatures.

T(K)	Pseudo-first-order model			Pseudo-second-order model		
	q_e_(mg/g)	K_1_(min^−1^)	R^2^	q_e_(mg/g)	K_2_(g.mg^−1^.min^−1^)	R^2^
298	65.38	0.1270	0.8782	171.8	0.007496	0.9983
308	41.84	0.1064	0.5991	166.7	0.01241	0.9987
318	30.53	0.1032	0.5971	161.6	0.01733	0.9999

**Table 6:** Coefficients of Weber-Morris model at different temperatures.

T(K)		298	308	318
Parameters	K_1’_(mg.g^−1^.min^−1/2^)	84.99	92.47	95.84
	R^2^	0.9927	0.9948	0.9956
	K_2’_(mg.g^−1^.min^−1/2^)	22.98	20.45	15.59
	R^2^	0.8625	0.8297	0.6620
	K_3’_(mg.g^−1^.min^−1/2^)	1.147	−0.01889	−0.1046
	R^2^	0.4927	0.3999	0.7391

## Effect of operating parameters on breakthrough curves

### Effect of feed flow rate

**Figure 8:**
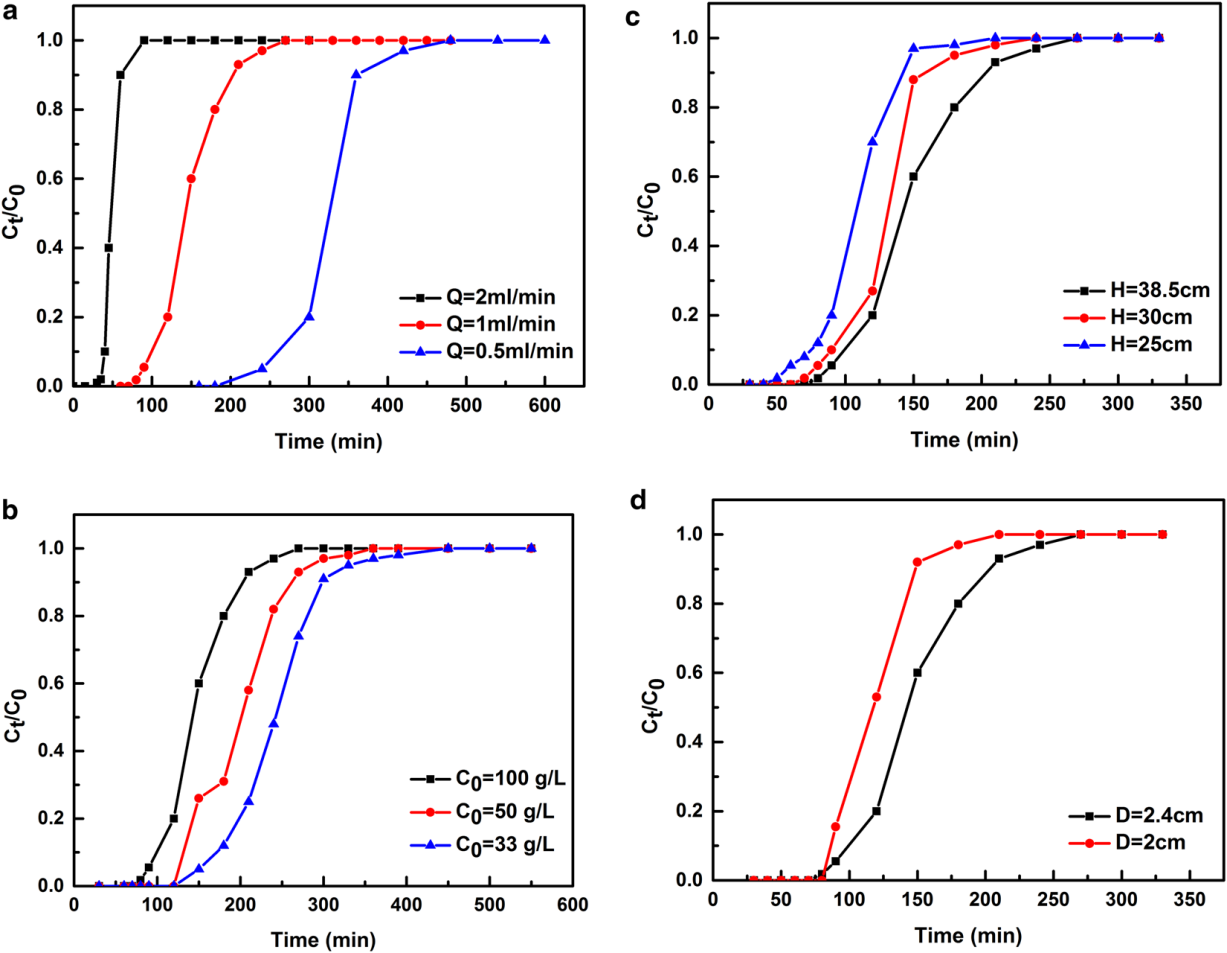
Breakthrough curves for citric acid adsorption on the fixed-bed column at different parameters (**a**) Different flow rate; (**b**) Different injection concentration; (**c**) Different column height; (**d**) Different column inner diameter.

Three feed flow rates (0.5, 1 and 2 mL/min) were performed while fixing the initial citric acid concentration of 100 g/L, the bed height of 38.5 cm and the bed inner diameter of 2.4 cm. The breakthrough curves with C_t_/C_0_ against time t shown in Fig. 8(a) become steeper at a higher flow rate. As shown in Table S3, the more adsorption capacity would be achieved at a slower flow rate. It could be explained by the decrease of the preserved time at high flow rate. This effect caused by higher perturbation was easier to weaken the interaction between adsorbate molecules and adsorbent particles, resulting in the decrease of adsorption^42,43^. However, considering the lower rate often accompanies excessive adsorption time and unnecessary instrumental spoilage, 1 mL/min will be more suitable from the practical operation point of view.

### Effect of initial concentration

The effects of initial concentrations on the citric acid adsorption behaviors were investigated at the feed concentration of 33, 50 and 100 g/L, respectively. The flow rate was remained at 1 mL/min, the bed height of 38.5 cm and the bed inner diameter of 2.4 cm. As the breakthrough curves depicted in Fig. 8(b), an interesting phenomenon could be observed that the retention time of the citric acid concentration profiles decreases with increasing the initial feed concentrations, namely the time was influenced by the initial concentrations, and it could be ascribed to the change of concentration gradient, which affected the saturation rate and breakthrough time.

### Effect of bed height

The citric acid breakthrough curves with bed heights of 38.5, 30 and 25 cm are described in Fig. 8(c) (the flow rate was 1.0 mL/min, bed inner diameter was 2.4 cm and feed concentration was 100 g/L). The retention time was reduced corresponding to the reduction of column height, whereas the loading capacities were almost identical (see Table S3) and it could be interpreted by the positive proportion between the retention time and column height. Reduction of the column height led to fewer adsorbent sits available loaded in the direction along the mobile phase moving, which shorten the retention of the adsorbate on the resins^44^. However, the loading capacity in terms of adsorption amount per mass of resins (mg/g) would be nearly unchanged.

### Effect of bed inner diameter

The citric acid breakthrough curves with bed inner diameters of 2 and 2.4 cm are presented in Fig. 8(d) with the bed height to be 38.5 cm, the feed flow rate to be 1 mL/min and the initial concentration to be 100 g/L, and the related characteristic parameters for each experimental breakthrough curve were given in Table S3. It was clear that the breakthrough time (t_b_) increased with increasing D_c_, which may owe to the fact that a larger number of resins existed along the cross sectional area of the column at a wider diameter column which was beneficial to the contact of citric acid molecules to the resins, resulting in a decrease of the mass transfer zone^44^.

Analyzing comprehensively these above results based on Table S3, the flowrate of 1 mL/min, the initial concentration of 100 g/L, the height of 38.5 cm, and the diameter of 2.4 cm would be the feasible manipulative parameters.

## Conclusions

During this study, we successfully tailored the pore textural property and surface functionality of the polymeric adsorbent to enhance its adsorption performance to citric acid through controlling the related parameters of the two continuous reactions (Friedel–Crafts alkylation and amino modification). The samples synthesized were characterized with N_2_ adsorption–desorption, FT-IR and analysis of residual chlorine content. Thereafter, the screened resin (named HM-65-2) was evaluated for adsorption of citric acid according to its adsorption capacity and adsorption selectivity. Both the Langmuir and Freundlich models correlate the equilibrium data well and the calculated thermodynamic parameters indicated that the adsorption process was a spontaneous and exothermic process. The adsorption kinetics of citric acid on the resin could be well correlated by the Pseudo-second-order model and the column dynamic adsorption experiments show that the elution flow rate, injection concentration, column height and inner diameter have significant effects on the adsorption performance of citric acid. Meanwhile, the force mechanism was explored from the molecular simulation perspective. It can be concluded that the adsorption behaviors of citric acid molecules on the methylamine-modified resins would be stronger.

Due to the unique properties in terms of proper adsorption capacities and high selectivity, HM-65-2 resins could be an attractive candidate in citric acid purification and further employed in the simulated moving bed separation process.

## Supplementary information

Supplementary Figure S1

Supplementary Figure S2

Supplementary Figure S3a

Supplementary Figure S3b

Supplementary Figure S3c

Supplementary Figure S3d

Supplementary Figure S4a

Supplementary Figure S4b

Supplementary information

## References

[1] Yang J, Webb AR, Ameer GA (2004). Novel Citric Acid-Based Biodegradable Elastomers for Tissue Engineering. Advanced Materials.

[2] Papagianni M, Michael M (2006). Morphological development of Aspergillus nigerin submerged citric acid fermentation as a function of the spore inoculum level. Application of neural network and cluster analysis for characterization of mycelial morphology. Microbial Cell Factories.

[3] Arai, Y., Sako, T. & Takebayashi, Y. *Material Processing Using Supercritical Fluids*. (2002).

[4] Ju H, Wei QF, Ren XL, Chen YX, Dong HS (2013). Study on Solvent Extraction of Citric Acid from Fermentation Broth. Advanced Materials Research.

[5] Luo GS, Shan XY, Qi X, Lu YC (2004). Two-phase electro-electrodialysis for recovery and concentration of citric acid. Separation and Purification Technology.

[6] Basu R, Sirkar KK (2004). Hollow fiber contained liquid membrane separation of citric acid. Aiche Journal.

[7] Zhong W, Li X, Yang H, Li E (2018). A novel, effective, and feasible method for deacidifying kiwifruit wine by weakly basic ion exchange resins. Journal of Food Process Engineering.

[8] Mudunkotuwa IA, Grassian VH (2010). Citric acid adsorption on TiO_2_ nanoparticles in aqueous suspensions at acidic and circumneutral pH: surface coverage, surface speciation, and its impact on nanoparticle-nanoparticle interactions. Journal of the American Chemical Society.

[9] Wu J, Peng Q, Arlt W, Minceva M (2009). Model-based design of a pilot-scale simulated moving bed for purification of citric acid from fermentation broth. Journal of Chromatography A.

[10] Gluszcz P, Jamroz T, Sencio B, Ledakowicz S (2004). Equilibrium and dynamic investigations of organic acids adsorption onto ion-exchange resins. Bioprocess & Biosystems Engineering.

[11] Juang RS, Chou TC (1996). Sorption of Citric Acid from Aqueous Solutions by Macroporous Resins Containing a Tertiary Amine Equilibria. Separation Science.

[12] Vega ED, Narda GE, Ferretti FH (2003). Adsorption of citric acid from dilute aqueous solutions by hydroxyapatite. Journal of Colloid and Interface Science.

[13] Lackovic K, Johnson BB, Angove MJ, Wells JD (2003). Modeling the adsorption of citric acid onto Muloorina illite and related clay minerals. Journal of Colloid & Interface Science.

[14] Li HY, Wang DF, Yu LN, Liu BJ, Xu Y (2009). Adsorption behavior of citric acid on resin of chitosan microspheres. Chinese Journal of Process Engineering.

[15] Sidorov SN (1999). Cobalt Nanoparticle Formation in the Pores of Hyper-Cross-Linked Polystyrene:  Control of Nanoparticle Growth and Morphology. Chemistry of Materials.

[16] Davankov VA, Rogoshin SV, Tsyurupa MP (2010). Macronet isoporous gels through crosslinking of dissolved polystyrene. Journal of Polymer Science Polymer Symposia.

[17] Tan L, Tan B (2017). Hypercrosslinked porous polymer materials: design, synthesis, and applications. Chemical Society Reviews.

[18] Bratkowska D (2010). Hydrophilic hypercrosslinked polymeric sorbents for the solid-phase extraction of polar contaminants from water. Journal of Chromatography A.

[19] Wang X, Huang J, Huang K (2010). Surface chemical modification on hyper-cross-linked resin by hydrophilic carbonyl and hydroxyl groups to be employed as a polymeric adsorbent for adsorption of p-aminobenzoic acid from aqueous solution. Chemical Engineering Journal.

[20] Ling X, Li H, Zha H, He C, Huang JH (2016). Polar-modified post-cross-linked polystyrene and its adsorption towards salicylic acid from aqueous solution. Chemical Engineering Journal.

[21] Kuang W, Li H, Huang J, Liu Y-N (2016). Tunable Porosity and Polarity of the Polar Hyper-Cross-Linked Resins and the Enhanced Adsorption towards Phenol. Industrial & Engineering Chemistry Research.

[22] Shao L, Li Y, Zhang T, Liu M, Huang J (2017). Controllable Synthesis of Polar Modified Hyper-Cross-Linked Resins and Their Adsorption of 2-Naphthol and 4-Hydroxybenzoic Acid from Aqueous Solution. Industrial & Engineering Chemistry Research.

[23] Wang X, Dai K, Chen L, Huang J, Liu Y-N (2014). An ethylenediamine-modified hypercrosslinked polystyrene resin: Synthesis, adsorption and separation properties. Chemical Engineering Journal.

[24] Guo Y (2018). A combined molecular dynamics simulation and experimental method to study the compatibility between elastomers and resins. Rsc Advances.

[25] Li C (2013). Chemical modification of Amberlite XAD-4 by carbonyl groups for phenol adsorption from wastewater. Chemical Engineering Journal.

[26] Cao H, Ji Y, Zhou J, Zhuang W, Wu J (2019). Competitive adsorption of vanillin and syringaldehyde on a macro-mesopore polymeric resin: modeling. Bioprocess and Biosystems Engineering.

[27] Vengatesan S, Santhi S, Jeevanantham S, Sozhan G (2015). Quaternized poly (styrene-co-vinylbenzyl chloride) anion exchange membranes for alkaline water electrolysers. Journal of Power Sources.

[28] Guanhua M (2007). Mechanism of oxidative reaction in the post crosslinking of hypercrosslinked polymers. European Polymer Journal.

[29] Du Y, George SM (2007). Molecular Layer Deposition of Nylon 66 Films Examined Using *in Situ* FTIR Spectroscopy. The. Journal of Physical Chemistry C.

[30] Fraser, C. G. & Auge, J. M. Physisorption of Gases, with Special Reference to the Evaluation of Surface Area and Pore Size Distribution. *Chemistry International–News magazine for IUPAC* (2011).

[31] Fu Z, He C, Huang J, Liu Y-N (2015). Polar modified post-cross-linked resin and its adsorption toward salicylic acid from aqueous solution: Equilibrium, kinetics and breakthrough studies. Journal of Colloid & Interface Science.

[32] Wang X (2013). Aniline modified hypercrosslinked polystyrene resins and their adsorption equilibriums, kinetics and dynamics towards salicylic acid from aqueous solutions. Chemical Engineering Journal.

[33] Traving M, Bart HJ (2002). Recovery of Organic Acids Using Ion-Exchanger-Impregnated Resins. Chemical Engineering & Technology.

[34] Souchon I, Rojas JA, Voilley A, Grevillot G (1996). Trapping of Aromatic Compounds by Adsorption on Hydrophobic Sorbents. Separation Science and Technology.

[35] Yan H, Du Q, Li A, Cheng R (2016). Efficient removal of chlorophenols from water with a magnetic reduced graphene oxide composite. Science China Chemistry.

[36] Ma X, Liu X, Anderson DP, Chang PR (2015). Modification of porous starch for the adsorption of heavy metal ions from aqueous solution. Food Chemistry.

[37] Wu J (2012). Separation of d-lactic acid from aqueous solutions based on the adsorption technology. Colloids & Surfaces A.

[38] Chiou MS, Li H (2003). Adsorption behavior of reactive dye in aqueous solution on chemical cross-linked chitosan beads. Chemosphere.

[39] Tu YJ, You C-F, Chang C-K, Chen M-H (2015). Application of magnetic nano-particles for phosphorus removal/recovery in aqueous solution. Journal of the Taiwan Institute of Chemical Engineers.

[40] Liu Y, Danyang Y, Luz S, Yanxue C, Xueyi L (2018). Adsorption of catechin onto cellulose and its mechanism study: Kinetic models, characterization and molecular simulation. Food Research International.

[41] Ho YS (2006). Review of Second-Order Models for Adsorption Systems. Cheminform.

[42] Monazam ER, Spenik J, Shadle LJ (2013). Fluid bed adsorption of carbon dioxide on immobilized polyethylenimine (PEI): Kinetic analysis and breakthrough behavior. Chemical Engineering Journal.

[43] Liu P, Zhang H, Xiang H, Yan Y (2016). Adsorption separation for high purity propane from liquefied petroleum gas in a fixed bed by removal of alkanes. Separation & Purification Technology.

[44] Mohammed N, Grishkewich N, Waeijen HA, Berry RM, Tam KC (2016). Continuous flow adsorption of methylene blue by cellulose nanocrystal-alginate hydrogel beads in fixed bed columns. Carbohydrate Polymers.

