# Supplementary figures and images for: Synthesis, adsorption and molecular simulation study of methylamine-modified hyper-cross-linked resins for efficient removal of citric acid from aqueous solution
Source: Sci Rep. 2020 Jun 15;10:9623. doi: 10.1038/s41598-020-66592-8 (PMC7295785; doi:10.1038/s41598-020-66592-8)

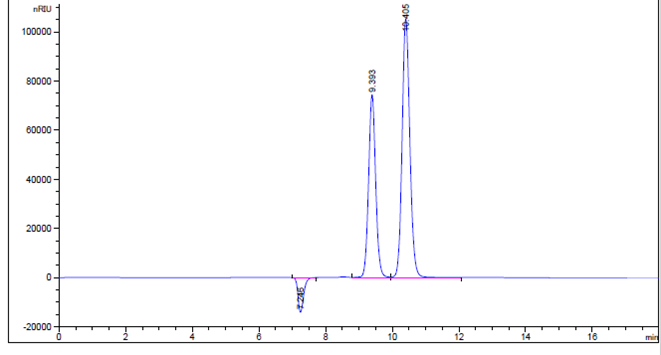

Supplement: Supplementary file 2 — Supplementary Figure S2 [file 41598_2020_66592_MOESM2_ESM.tif]
