# Supplementary material for: Synthesis, adsorption and molecular simulation study of methylamine-modified hyper-cross-linked resins for efficient removal of citric acid from aqueous solution
Source: Sci Rep. 2020 Jun 15;10:9623. doi: 10.1038/s41598-020-66592-8 (PMC7295785; doi:10.1038/s41598-020-66592-8)
Supplement: Supplementary file 9 — Supplementary information [file 41598_2020_66592_MOESM9_ESM.doc]

**Synthesis, adsorption and molecular simulation study of methylamine-modified hyper-cross-linked resins for efficient removal of citric acid from aqueous solution**

Xiaoqiang Peng ^abc^, Pengpeng Yang^abc^, Kun Dai^abc^, Yong Chen^abc^, Xiaochun Chen^abc^, Wei Zhuang^abc^, , Hanjie Ying^*abcd^ and Jinglan Wu^*abc^

### Analytical method

The standard curves of citric acid and glucose for concentration detection (under 10 g/L) and chromatogram were shown in Fig. S1 and S2, respectively. Table S1 lists the correlation coefficients of the standard curves.

**Table S1.**  Correlation coefficients of the standard curves in HPLC

|  | Slope | R^2^ |
| --- | --- | --- |
| Citric acid |  | 0.9978 |
| Glucose |  | 0.9974 |

## Characterization of methylamine-modified hyper-cross-linked resins

**Table S2.** All the necessary BET and mainly elemental analysis data in different resins.

|  | Special surface area（m^2^/g） | Pore volume  （cm^3^/g） | Chlorine content  （%） | Nitrogen content  （%） |
| --- | --- | --- | --- | --- |
| precursor | 31.88 | 0.0830 | 18.68 | / |
| H-65-1 | 651.3 | 0.4656 | 6.31 | / |
| H-65-2 | 776.7 | 0.5482 | 5.54 | / |
| H-65-4 | 762.7 | 0.5447 | 5.45 | / |
| H-85-1 | 829.4 | 0.5761 | 5.42 | / |
| H-85-2 | 884.2 | 0.5934 | 3.53 | / |
| H-85-4 | 946.1 | 0.6248 | 3.20 | / |
| HM-65-1 | 513.5 | 0.4083 | 0.94 | 5.28 |
| HM-65-2 | 656.4 | 0.4729 | 0.90 | 4.55 |
| HM-65-4 | 673.1 | 0.4629 | 0.63 | 4.39 |
| HM-85-1 | 626.9 | 0.4052 | 0.79 | 4.51 |
| HM-85-2 | 658.3 | 0.4258 | 0.68 | 2.87 |
| HM-85-4 | 746.5 | 0.4487 | 0.53 | 2.24 |

## Comparison between weak basic anion-exchanger and HM-65-2 resin

The compare experiments of adsorption capacities under various pH solutions were carried out to illuminate the tailor-made methylamine-modified resin superiority. In this part a kind of commercial available weak basic anion-exchanger (Amberlite FPA53) was chosen for adsorbing citric acid under different pH solutions and hot water (65 ^o^C) was chose as an eluent. The solution pH was adjusted by HCl/NaOH. The citric acid adsorption experiments in HM-65-2 resin under different pH solutions were also accomplished. The desorption rate *D* (%) was calculated by following formula:


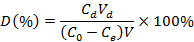


where *C_d_* (g/L) is the concentration of citric acid in solution after desorption and *V_d_* (mL) is the volume of desorbent.

The adsorption experimental results of the weak basic anion-exchanger and the HM-65-2 resin were shown in Figure S4, respectively. Obviously, the adsorption capacity of citric acid was significantly affected by pH value in solution. When the pH of the solution is not adjusted by HCl/NaOH, the adsorption capacity of citric acid in the weak anion-exchange resin reached the maximum value (378.1mg/g), which was higher than that of the HM-65-2 resin (136.3mg/g). This enormous difference could be attributed to the greater interaction between the anion-exchange resin and citric acid．However, the desorption rates of the weak basic anion-exchange resin were all lower than 40%, which was much lower than that of the HM-65-2 resin. It indicates that a “green” separation routine can be probably established with the tailor-made HM-65-2 resin by adsorption of citric acid and elution it with only hot water.

## Effect of operating parameters on the breakthrough curves

The breakthrough capacities (*q_b_*) as well as the saturated capacities (*q_s_*) of citric acid under various operating conditions were listed in Table S3.

**Table S3.** Characteristic parameters of citric acid breakthrough curves under various operating conditions.

| Q(mL/min) | H(cm) | D(cm) | C_0_(g/L) | q_b_(mg/g) | q_s_(mg/g) |
| --- | --- | --- | --- | --- | --- |
| 2.00 | 38.50 | 2.40 | 100.0 | 58.08 | 83.42 |
| 1.00 | 38.50 | 2.40 | 100.0 | 74.62 | 123.6 |
| 0.50 | 38.50 | 2.40 | 100.0 | 99.38 | 134.1 |
| 1.00 | 38.50 | 2.40 | 50.00 | 60.88 | 82.69 |
| 1.00 | 38.50 | 2.40 | 33.00 | 41.04 | 65.79 |
| 1.00 | 30.00 | 2.40 | 100.0 | 75.76 | 122.4 |
| 1.00 | 25.00 | 2.40 | 100.0 | 66.16 | 119.7 |
| 1.00 | 38.50 | 2.00 | 100.0 | 91.05 | 121.3 |
